# Determination of Phytoestrogen Content in Fresh-Cut Legume Forage

**DOI:** 10.3390/ani6070043

**Published:** 2016-07-14

**Authors:** Pavlína Hloucalová, Jiří Skládanka, Pavel Horký, Bořivoj Klejdus, Jan Pelikán, Daniela Knotová

**Affiliations:** 1 Department of Animal Nutrition and Forage Production, Faculty of Agronomy, Mendel University in Brno, Zemědělská 1/1665, Brno CZ 613 00, Czech Republic; jiri.skladanka@mendelu.cz (J.S.); pavel.horky@mendelu.cz (P.H.); 2 Department of Chemistry and Biochemistry, Faculty of Agronomy, Mendel University in Brno, Zemědělská 1/1665, Brno CZ 613 00, Czech Republic; borivoj.klejdus@mendelu.cz; 3 Research Institute for Fodder Crops, Ltd., Troubsko, Zahradní 1, Troubsko 664 41, Czech Republic; pelikan@vupt.cz (J.P.); knotova@vupt.cz (D.K.)

**Keywords:** phytoestrogens, *Trifolium*, *Medicago*, isoflavones, biochanin A, formononetin

## Abstract

**Simple Summary:**

Phytoestrogens comprise a group of substances negatively influencing the development and function of animal reproductive organs. Their appearance in forage crops can reduce feeding values, cause dietary disorders, and lead to animal health damage. This study evaluated the occurrence of individual phytoestrogens in various species of annual and perennial legumes and their levels in dry forage. It appeared that feeding large amounts of red clover presents a potential risk, but red clover can be replaced with the annual Persian clover, in which markedly lower phytoestrogen levels were detected.

**Abstract:**

The aim of the study was to determine phytoestrogen content in fresh-cut legume forage. This issue has been much discussed in recent years in connection with the health and safety of feedstuffs and thus livestock health. The experiments were carried out on two experimental plots at Troubsko and Vatín, Czech Republic during June and July in 2015. Samples were collected of the four forage legume species perennial red clover (variety “Amos”), alfalfa (variety “Holyně”), and annuals Persian clover and Alexandrian clover. Forage was sampled twice at regular three to four day intervals leading up to harvest and a third time on the day of harvest. Fresh and wilted material was analyzed using liquid chromatography–mass spectrometry (LC-MS). Higher levels (*p* < 0.05) of isoflavones biochanin A (3.697 mg·g^−1^ of dry weight) and formononetin (4.315 mg·g^−1^ of dry weight) were found in red clover than in other species. The highest isoflavone content was detected in red clover, reaching 1.001% of dry matter (*p* < 0.05), representing a risk for occurrence of reproduction problems and inhibited secretion of animal estrogen. The phytoestrogen content was particularly increased in wilted forage. Significant isoflavone reduction was observed over three to four day intervals leading up to harvest.

## 1. Introduction

Phytoestrogens are non-steroidal substances of plant origin. Their essential characteristic is to evoke variously strong estrogenic effects. Most of the products derive from phenylpropane metabolism and differ from mammalian estrogen [1,2,3]. The isoflavones genistein and daidzein are among the most active phytoestrogens. They can be found mainly in legumes, such as clover [1]. Comparable amounts of isoflavonoids can be detected in clover’s roots, stems, and leaves but smaller quantities in flowers. In red clover, primarily the four isoflavones genistein, daidzein, biochanin A, and formononetin are found. By comparison, soybean contains only two of these substances: daidzein and genistein [1].

**Figure 1:**
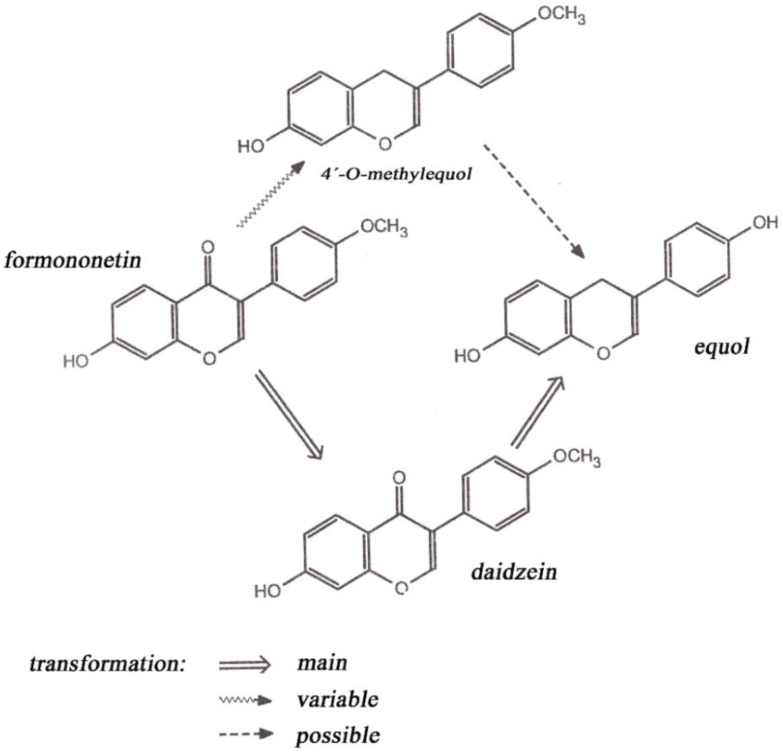
Formononetin metabolism in sheep.

Biochanin A (a 4’-methoxy derivative of genistein) has been shown to produce chemopreventive effects against various types of cancer (e.g., prostate, breast, colon) [4]. Formononetin (a 4′-methoxy derivative of daidzein) is a precursor of daidzein. Biochanin A and formononetin are methylated precursors occurring in the human body and probably are metabolized in the colon on the practically most important phytoestrogens genistein (4′,5,7-trihydroxyisoflavone) and daidzin (4′,7-dihydroxyisoflavone) [1]. Some sources claim that formononetin in ruminants is estrogenically more active than is genistein degraded to daidzein and then metabolized to equol, which is a weak estrogenic compound (Figure 1) [5]. Although formononetin itself shows no estrogenic activity, its metabolite causes so-called clover disease occurring mainly in small ruminants (especially sheep). Estrogenic activity of isoflavones is approximately 10^−3^ to 10^−5^ of the diethylstilbestrol equivalent. This synthetic nonsteroidal estrogen was formerly used as a growth promoter in animals, but today it is classified as a carcinogen [6]. Coumestrol occurs in soybean, red clover and alfalfa, among other plants [7]. The occurrence of coumestrol may be evoked by leaf disease [5]. In New Zealand, a reduction of reproductive performance was observed in ewes on pastures including some alfalfa in the stand. The reason for this was determined to be the occurrence of coumestans induced by leaf diseases [8].

**Figure 2:**
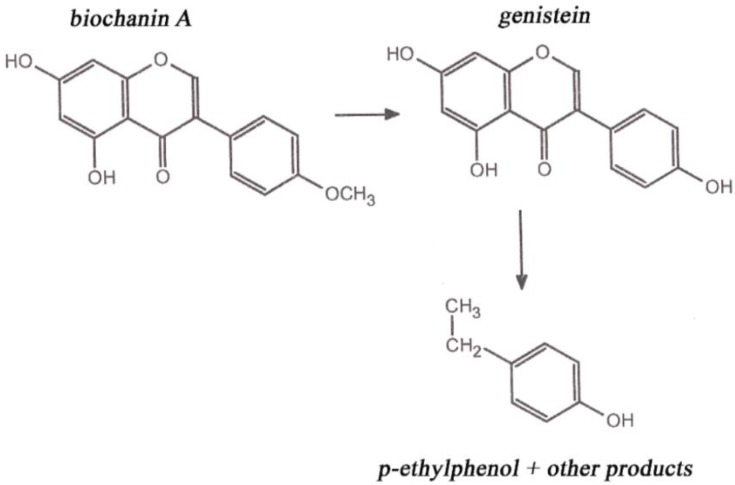
Biochanin A metabolism in sheep.

In the animal body, phytoestrogens inhibit secretion of animal estrogens and become more active substances inasmuch as they have the character of progesterone. In sheep, biochanin A and genistein are degraded to p-ethylphenol and phenolic acid (Figure 2). Such metabolites are absorbed by the rumen wall into the blood and act as endogenous hormones in the bodies of sheep. Levels of 3–5 mg·L^−1^ of equol in blood plasma can cause infertility in sheep grazed on clover stands. Disruptions of ovulation, false estrus, and degradation of genitalia are among the effects in animals. Mammary glands are affected most by formononetin. When feeding alfalfa, there is higher incidence of mastitis and heifers may exhibit milk secretion [6].

Phytoestrogens are abundantly found in clover but also in *Dactylis glomerata*, perennial ryegrass, and dandelion. In conditions of stress, phytoestrogen content can be increased. The estrogenic activity is very weak. Some phytoestrogens exert an agonistic effect on the isoform of the estrogen receptor known as Erβ, which mainly occurs in tissues of short bones [2,3]. The available sources indicate that phytoestrogen values exceeding 1% of dry matter (DM) are associated with reproductive disorders in animals. It is reported that phytoestrogen values remain below that threshold in white clover [5]. The content of isoflavonoids is said to range from 1% to 2% of DM in red clover [1]. The issue of phytoestrogens in feed is a current topic especially in ruminant nutrition, and identification of clovers containing estrogenic compounds can help to overcome the limitations in feeding this forage [8].

The aims of the present study are to evaluate the health and safety of forage with reference to the contents of isoflavones (biochanin A, formononetin, genistein, ononin, sissotrin, daidzein, and daidzin), to compare the health and safety of forage consisting of perennial (red clover and alfalfa) and annual (Alexandrian clover and Persian clover) forage legumes, as well as to assess the impact of wilting on the content of isoflavones.

## 2. Materials and Methods

### 2.1. Experimental Plots

The experimental stands were established at the Forage Research Station in Vatín, Czech Republic, and at the Research Institute for Fodder Crops, Ltd., Troubsko, Czech Republic. The experimental plot at Vatín is located at an altitude of 650 m a.s.l. It has average annual rainfall of 660 mm and average annual temperature of 7.2 °C. The experimental plot at Troubsko is situated at altitude 277 m a.s.l. It has average annual rainfall of 543 mm and average annual temperature of 8.3 °C [9]. Soil testing at Vatín showed the content of nutrients available for annual and perennial forage legumes to be 128.21 mg·kg^−1^ of P, 396.10 mg·kg^−1^ of K, 1694 mg·kg^−1^ of Ca, and 326.60 mg·k^−1^ of Mg. Soil pH was 6.14. In Troubsko, the content of nutrients available for annual clovers was 75.5 mg·kg^−1^ of P, 216.3 mg·kg^−1^ of K, 2965.25 mg·kg^−1^ of Ca, and 346.20 mg·kg^−1^ of Mg. The pH was 5.69. For perennials at Troubsko, soil testing detected 37.3 mg·kg^−1^ of P, 152.95 mg·kg^−1^ of K, 6548 mg·kg^−1^ of Ca, 206.45 mg·kg^−1^ of Mg, and pH of 7.33.

### 2.2. Experimental Design

The stands of perennial forage legumes were established in the spring of 2013 (biomass of the second harvest year was evaluated). The stands of annual clovers were established in the spring of 2015. The small-plot experiments were set up in three replications on plots measuring 1.25 × 8 m. The first evaluated factor was species, consisting of alfalfa (*Medicago sativa* L.; variety “Holyna”), red clover (*Trifolium pratense* L. (4n); variety “Amos”), Alexandrian clover (*Trifolium alexandrinum* L.), and Persian clover (*Trifolium resupinatum* L.). The second evaluated factor was sampling, with levels of Sampling 1, Sampling 2, and Sampling 3. The data were obtained from two experimental plots identified as Vatín and Troubsko. An additional evaluated factor was forage wilting, with categories of fresh-cut forage (unwilted) and wilted forage.

**Figure 3:**
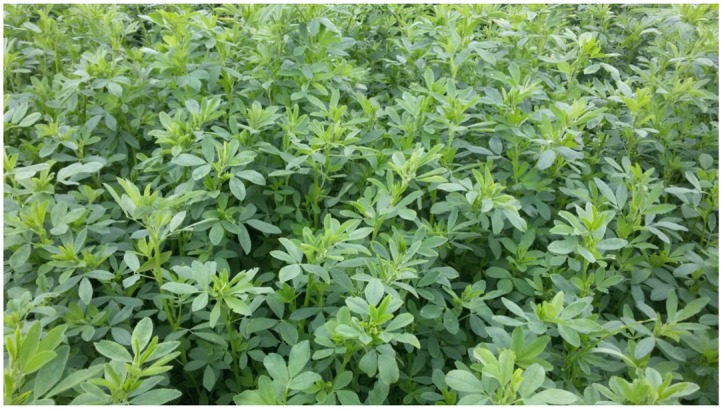
*Medicago sativa* L. growing on the experimental plot.

Alfalfa (*M. sativa*) (Figure 3) produces high nutritional value and biomass. Alfalfa is more efficient in terms of reducing soil erosion than are annual forage legumes, and therefore it is widely used in pastures. Highly adaptable to different environments, alfalfa is grown in Europe, North America, Australia, and China [10].

**Figure 4:**
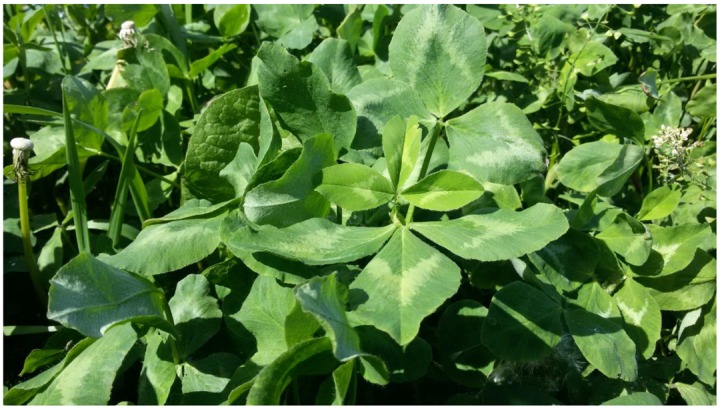
*Trifolium pratense* L. growing on the experimental plot.

Red clover (*T. pratense*) (Figure 4) is a perennial forage crop very well suited for pasture utilization because it provides high-quality and digestible protein. Deep roots allow growth even during summer and in times of drought. It contains a large number of known phytoestrogens. For this reason, extracts from clover are used in traditional and folk medicines as an alternative to hormone replacement therapy for menopausal symptoms and other illnesses [11,12].

**Figure 5:**
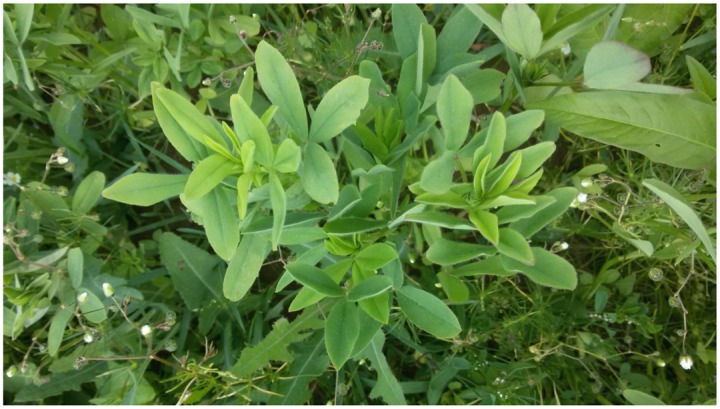
*Trifolium alexandrinum* L. growing on the experimental plot.

Alexandrian clover (*T. alexandrinum*) (Figure 5) is an annual forage crop widely grown in the countries of the Middle East, where it originated. Favored for its high yield of biomass, crude protein content, and fixation of atmospheric nitrogen, Alexandrian clover is commonly used to feed livestock or as a green manure crop. In Egypt, it is the main feed for grazing animals [13]. With respect to its yield and crude protein content, there is contemplation that Alexandrian clover might also be sown in central European conditions.

**Figure 6:**
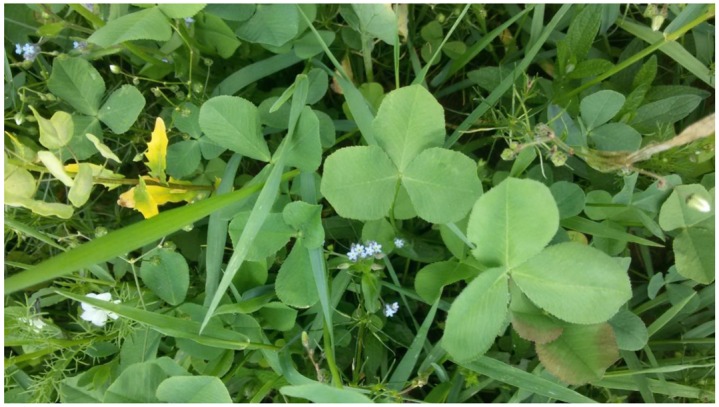
*Trifolium resupinatum* L. growing on the experimental plot.

Persian clover (*T. resupinatum*) (Figure 6) is an annual grazing forage originating from the Mediterranean region, where it has been commonly cultivated for several centuries. At higher altitudes, Persian clover has been grown since the beginning of the 20th century. Despite such a long period of Persian clover’s cultivation, secondary metabolites potentially affecting the nutritional value of the biomass have not been explored in detail [14].

### 2.3. Stand Treatment

Three cuttings were taken from the stands. The biomass of the first growth was taken in 2015 for the evaluation of phytoestrogens. The forage was harvested three times at intervals of three to foour days, such that, by the last term, the forage was in the late-bud phase (Table 1). The stands were not fertilized. The harvest was taken using a self-propelled small-plot mower and leaving a stubble height of 0.07 m.

### 2.4. Sampling and Sample Preparation

A first set of 500 g samples was frozen immediately after harvest. A second set of 500 g samples was left to wilt naturally for 24 h (in shade under laboratory conditions, temperature 22 °C, relative humidity 70%) and subsequently frozen. Immediately before analysis, the samples were dried at 55 ± 3 °C and then milled to 0.1 mm particle size using a laboratory mill. A 50 mg sample of the material thus prepared was combined in a mortar with 1 mL of 80% methanol and a pinch of inert sea sand to prepare extractions. The resulting mixture was centrifuged and the supernatant filtered through a nylon syringe filter (13 mm in diameter, 0.45 µm pore size).

### 2.5. Conditions for Liquid Chromatography-Mass Spectrometry (LC-MS)

The separation of isoflavones was made in an Agilent Technologies 1200 Series liquid chromatograph (Santa Clara, CA, USA). A Zorbax Poroshell 120 EC-18 column (Santa Clara, CA, USA) with dimensions 3.0 × 50 mm and particle size 2.7 μm was used. The mobile phase consisted in the following: solvent A = acetonitrile, solvent B = 0.2% acetic acid (*v*/*v*). A linear gradient was selected consisting of a mobile phase at time 0 min t0 = 85% of B, t0.30 = 40% of B, t1.40 = 0% of B, and t1.8 = 85% of B. The flow rate for the mobile phase was 0.7 mL·min^−1^. Temperature was 60 °C at the column thermostat. Parameters for mass spectrometry were the following: sheath gas temperature 350 °C, sheath gas flow rate 13 L·min^−1^, nebulizer pressure 50 psi, nozzle temperature 400 °C, and gas flow 12 L·min^−1^. Chamber voltage was set at 4 kV. Mass spectra were monitored in negative mode.

Retention times and regression equations were published in Klejdus et al. [15]. Briefly, all regression coefficients were 0.9998. Limits of detection (LODs) and limits of quantification (LOQs) varied within the range from 0.06 to 1.81 and from 0.19 to 6.02 ng/mL, respectively. The total time for analysis of a sample was approximately 15 min.

### 2.6. Statistical Evaluation

The data were statistically analyzed using STATISTICA.CZ version 10.0 (StatSoft, Prague, Czech Republic). The results were expressed as mean ± standard deviation. Statistical significance between group species was tested using ANOVA and Scheffé’s post hoc test. The multifactor analysis examined the parameters of study plot, species, wilting, and sampling number. Statistically significant differences were considered to exist at *p* < 0.05.

## 3. Results

**Figure 7:**
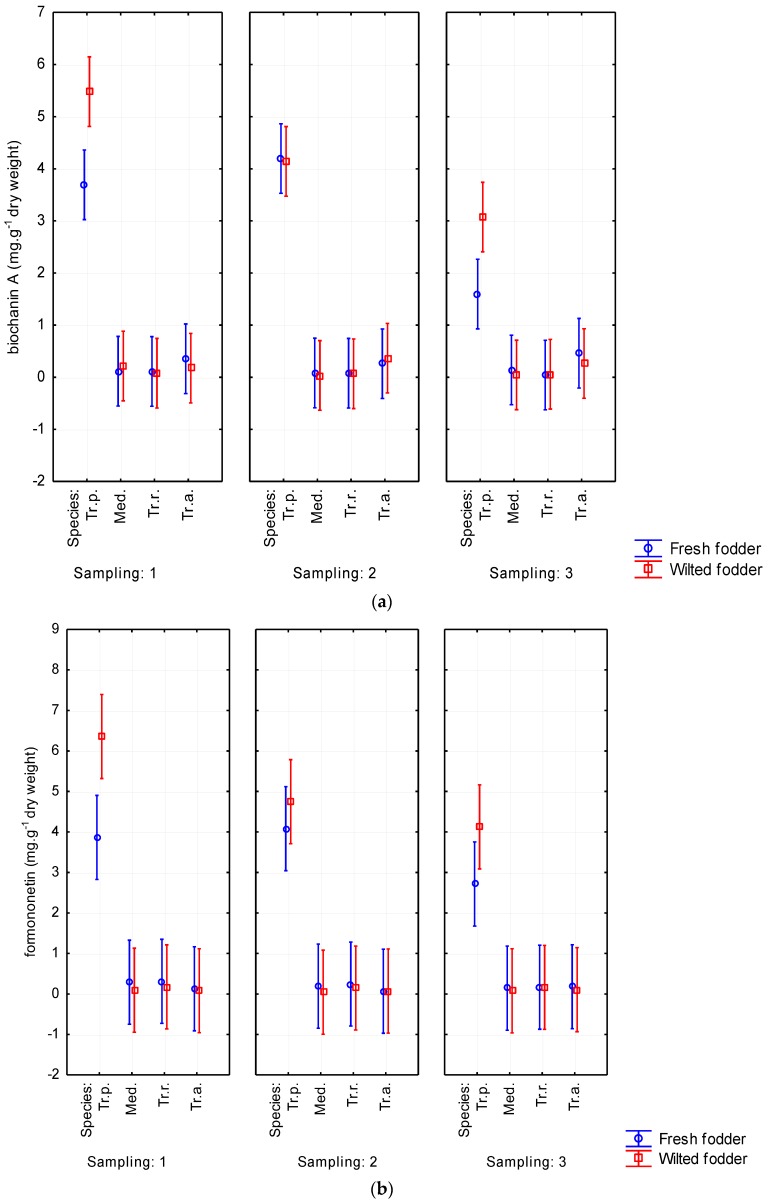

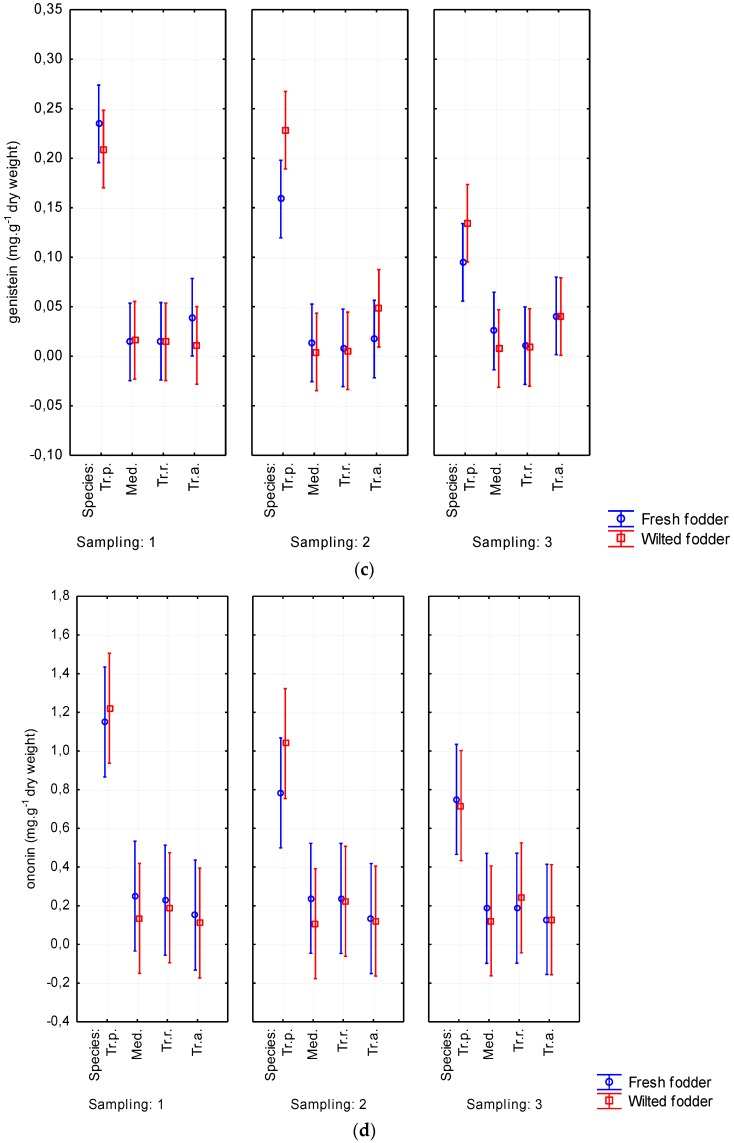

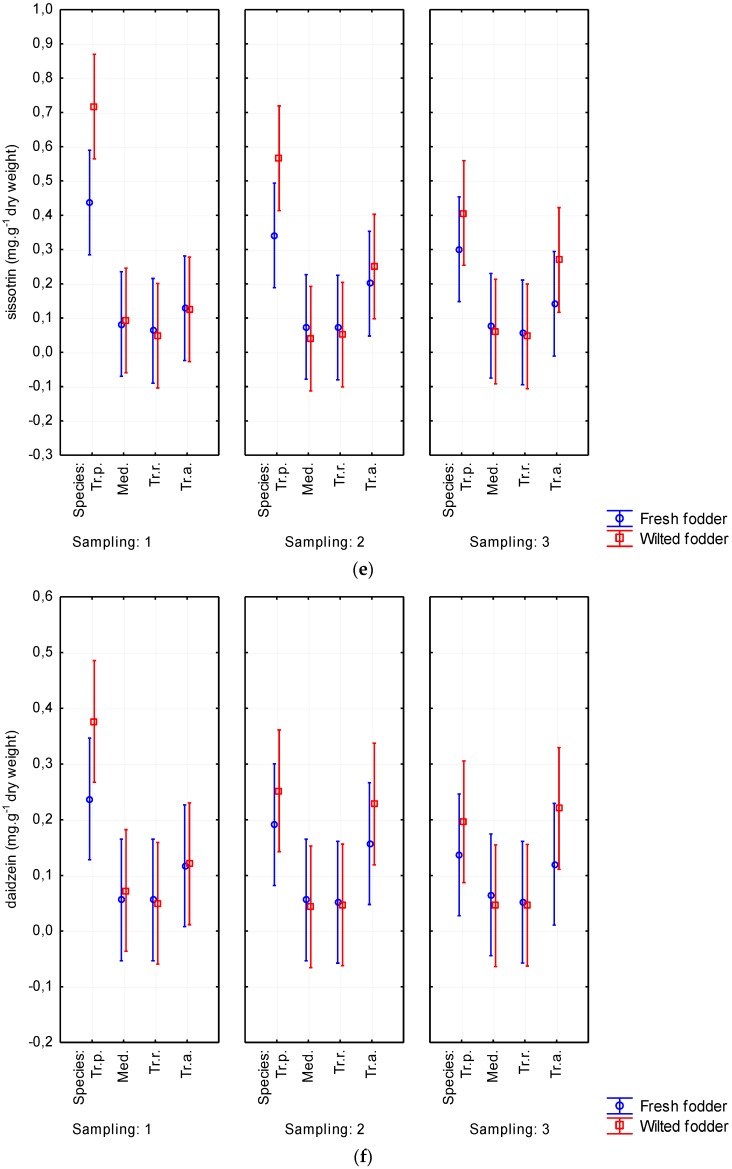

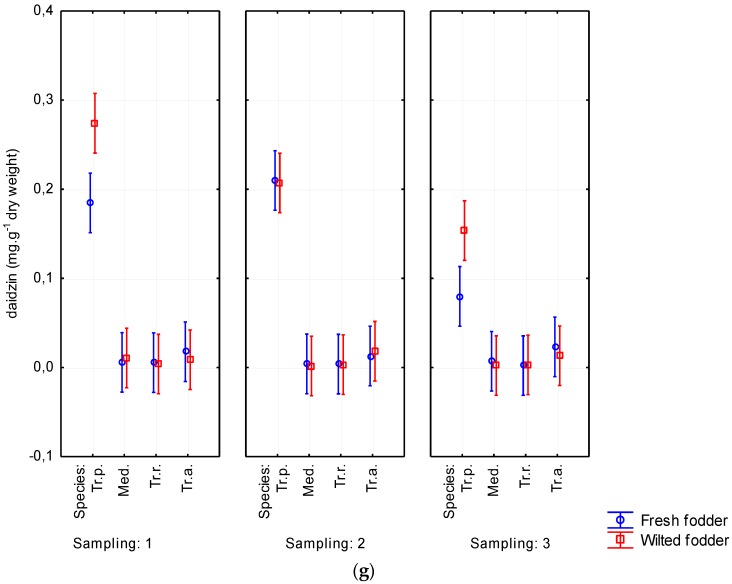
Wilting’s influence on contents of biochanin A (**a**); formononetin (**b**); genistein (**c**); ononin (**d**); sissotrin (**e**); daidzein (**f**); and daidzin (**g**) from Sampling 1 to Sampling 3 (mg·g^−1^ of dry matter) in *Medicago sativa* (Med.), *Trifolium pratense* (Tr.p.), *Trifolium resupinatum* (Tr.r.), and *Trifolium alexandrinum* (Tr.a.). Error bars indicate 95% confidence interval.

Mainly biochanin A and formononetin were detected. Depending on forage plant species, the values of biochanin A ranged from 0.072 to 3.696 mg·g^−1^ of dry weight. The content of formononetin reached 4.315 mg·g^−1^. Furthermore, ononin (0.129–0.943 mg·g^−1^ of dry weight) was also represented. The results from analyzing all phytoestrogens in fresh and wilted forage are shown in Table 2. The highest content of isoflavones was detected on red clover, reaching of 1.001% of DM (*p* < 0.05). Conversely, Persian clover showed the lowest content of all evaluated isoflavones (Table 2 and Table 3). Alexandrian clover had higher content of some isoflavones (biochanin A, sissotrin, daidzein) than did alfalfa. A tendency was observed for phytoestrogen content to increase due to wilting. Biochanin A content was determined up to 1.166 mg·g^−1^ of DM in wilted forage while it reached only 0.928 mg·g^−1^ of DM in fresh-cut forage. Similarly, formononetin was noticed to be higher in wilted forage (1.349 mg·g^−1^ of DM) than in fresh-cut forage (1.033 mg·g^−1^ of DM). The increasing content of isoflavones due to wilting was evident without regard to harvest date (Figure 7). The isoflavone content decreased with aging of plants. This tendency was particularly apparent for biochanin A and formononetin, for which the content decreased from 1.278 to 0.710 mg·g^−1^ of DM and from 1.413 to 0.960 mg·g^−1^ of DM, respectively. The total content of phytoestrogens ranged from 0.056% to 1.001% of DM in the studied forage crops (Table 3).

## 4. Discussion

In recent years, phytoestrogens have been investigated by Wu et al. [16], Lucas et al. [5] and Barreira et al. [17]. These works are primarily directed at evaluating such traditional legume species as red clover and alfalfa. Other clover species have their places in animal nutrition as well. Assessment of phytoestrogen content in Persian clover and Alexandrian clover will become more important as we include more of these species into animal diets.

Phytoestrogens content had been evaluated by Lucas et al. in a similar experiment [5]. The two isoflavones genistein and formononetin were measured. The average values for formononetin were found to be 125.9 µg·g^−1^ of DM (in wild forms) and 110.4 µg·g^−1^ of DM (in transgenic cultivars). The respective average values for genistein were 24.3 and 23.6 µg·g^−1^ of DM. Considerable variance was observed among the plants. Wu et al. [16] had measured very low concentrations of biochanin A and daidzein in blooms of red clover, and in the work of Lucas et al. [5], these phytoestrogens (biochanin A and daidzein) were not detected.

**Figure 8:**
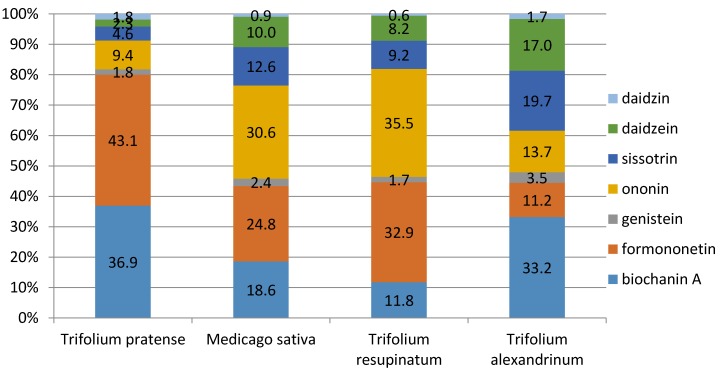
Percentages of isoflavones determined in *Trifolium pratense*, *Medicago sativa*, *Trifolium resupinatum*, and *Trifolium alexandrinum*.

According to the results of Nykänen-Kurki et al. [18], formononetin constituted nearly 90% of all phytoestrogens in red clover while genistein contributed another 5–10%. As shown in Figure 8, the percentage representation of these two substances, in particular, was lower in our experiment, with formononetin ranging from 11.2% to 43.1%, depending upon species.

Kwiatkowska [7] reported that the values for formononetin can reach 13.22 mg·g^−1^ in red clover and 17.71 mg·g^−1^ in alfalfa. Barreira et al. [17] related that formononetin content ranges from 0.011 to 2.8 mg·g^−1^, depending upon the individual *Medicago* species.

Rijke et al. [19] also determined isoflavone values for red clover in their work. According to their findings, the contents of individual substances are as follows: biochanin A—0.33 mg·g^−1^, formononetin—0.6 mg·g^−1^, sissotrin—0.54 mg·g^−1^, ononin—0.97 mg·g^−1^, and daidzin—0.042 mg·g^−1^. In their experiment, daidzein and genistein were not detected in red clover leaves. Their values for sissotrin and ononin are very similar to the values measured in our experiment and reported in Table 2. The contents of other isoflavones determined in our extracts are higher—up to 4.315 mg·g^−1^ in the case of formononetin. These widely differing values can be due to differences either in experimental methods or climatic conditions.

Ramírez-Restrepo and Barry [8] found the total content of isoflavones in red clover forage to range from 7 to 14 mg·g^−1^ of DM. As can be seen in Table 2 and Table 3, our measurements confirm their findings.

Barreira et al. [17] found that the average content of all isoflavones ranged from 1.25 to 3.16 mg·g^−1^ of DM in *Medicago* species, which was significantly higher than our measurements. The variance could be due to differences between the experiments. Whereas Barreira et al. evaluated nine species of *Medicago*, only *M. sativa* was included in our measurement. Lucas et al. [5] also reported that phytoestrogen values exceeding 1% of DM could be connected with animal health problems. In the animal body, phytoestrogens inhibit secretion of animal estrogens and become more active substances. In sheep, biochanin A and genistein are degraded to p-ethylphenol and phenolic acid. Such metabolites are absorbed by the rumen wall into the bloodstream and act as endogenous hormones in the sheep’s bodies [6]. This critical value was exceeded (at 1.001%) in our experiment’s red clover samples. This is associated with a risk for increased level of equol in blood plasma and possibilities for reproductive problems to occur in animals fed such forage. Feeding perennial red clover can be risky if fed as only a feedstuff to ruminants, such as by pasturing on a red clover monoculture [6]. Another factor in limiting the feeding of red clover can be the heightened proportion of formononetin (43.1%) within the overall isoflavone content. According to Lucas et al. [5], formononetin is estrogenically more active in ruminants than is, for example, genistein. The feeding of red clover need not necessarily constitute an increased risk of health problems if, for example, it is included into a total mixed ration and the other feedstuffs in the ration do not themselves contain excessive phytoestrogens.

The overall content of isoflavones in the samples of Persian clover, at 0.0614%, did not exceed the critical value of 1% of DM. Its growth in Central European conditions may provide a certain substitute for red clover in livestock nutrition. The values for individual isoflavones measured in Persian clover proved to be close to those for alfalfa and represented no such health risk as exists in feeding red clover.

Alexandrian clover also did not represent a potential risk for health problems from its use as a feedstuff in terms of total isoflavones (0.0944%) or in the DM representation of individual isoflavones (33.2% biochanin A, 19.7% sissotrin, and 17% daidzein). The greatest occurrence of genistein—as high as 3.5%—was determined in the samples of Alexandrian clover (Figure 8). According to Kašparová (2013) [1], genistein together with daidzein are the most important of all isoflavones.

According to our measured results for the phytoestrogens examined here, the values for biochanin A, formononetin, genistein, daidzein, sissotrin, ononin, and daidzin do not exceed the critical value of 1% of DM. A potential risk for livestock can be presented, however, by the concentration of isoflavones in red clover, which were measured at 1.001% of DM.

## 5. Conclusions

The greatest isoflavone content was found in red clover. In the forage generally, biochanin A and formononetin were mainly present. Other isoflavones detected were genistein, ononin, sissotrin, daidzein, and daidzin. Although the lowest amounts of isoflavones occurred in Persian clover, it can be said from the viewpoint of statistical evaluation that the contents of isoflavones in Persian clover, Alexandrian clover, and alfalfa were comparable. There was a clear tendency for isoflavone content to increase due to wilting. There was a strong influence on phytoestrogens content due to differences between species. By their proportions of individual substances, the perennial alfalfa and annual Persian clover proved to be very similar, and the processing of the harvested forage could also affect these. Wilted forage was shown to represent a potential feeding risk in comparison to fresh-cut forage due to its increased content of anti-nutritional substances.

## Figures and Tables

**Table 1:** Sampling terms and phenophase for individual species.

Sampling Order	*Trifolium pratense* L.	*Medicago sativa* L.	*Trifolium resupinatum* L.	*Trifolium alexandrinum* L.
**Vatín Experimental Plot**				
Sampling 1	2 June 2015 late vegetative	2 June 2015 late vegetative	24 June 2015 late vegetative	24 June 2015 late vegetative
Sampling 2	5 June 2015 early bud	5 June 2015 early bud	27 June 2015 early bud	27 June 2015 early bud
Sampling 3	8 June 2015 late bud	8 June 2015 late bud	30 June 2015 late bud	30 June 2015 late bud
**Troubsko Experimental Plot**				
Sampling 1	28 May 2015 late vegetative	28 May 2015 late vegetative	19 June 2015 late vegetative	19 June 2015 late vegetative
Sampling 2	1 June 2015 early bud	1 June 2015 early bud	22 June 2015 early bud	22 June 2015 early bud
Sampling 3	4 June 2015 late bud	4 June 2015 late bud	25 June 2015 late bud	25 June 2015 late bud

**Table 2:** Content of biochanin A, formononetin, genistein, ononin, sissotrin, daidzein, and daidzin in dry matter of *Trifolium pratense*, *Medicago sativa*, *Trifolium resupinatum*, and *Trifolium alexandrinum* (mg·g^−1^).

Factor	*N*	Content of Isoflavones mg·g^−1^						
		Biochanin A	Formononetin	Genistein	Ononin	Sissotrin	Daidzein	Daidzin
Species								
*Trifolium pratense* L.	12	3.697 ± 1.40 ^a^	4.315 ± 1.55 ^a^	0.177 ± 0.06 ^a^	0.943 ± 0.35 ^a^	0.462 ± 0.20 ^a^	0.232 ± 0.11 ^a^	0.185 ± 0.07 ^a^
*Medicago sativa* L.	12	0.105 ± 0.11 ^b^	0.141 ± 0.12 ^b^	0.014 ± 0.01 ^b^	0.173 ± 0.07 ^b^	0.071 ± 0.02 ^b^	0.057 ± 0.02 ^b^	0.005 ± 0.01 ^b^
*Trifolium resupinatum* L.	12	0.072 ± 0.03 ^b^	0.202 ± 0.07 ^b^	0.011 ± 0.01 ^b^	0.218 ± 0.04 ^b^	0.057 ± 0.01 ^b^	0.051 ± 0.01 ^b^	0.004 ± 0.00 ^b^
*Trifolium alexandrinum* L.	12	0.314 ± 0.20 ^b^	0.106 ± 0.06 ^b^	0.033 ± 0.02 ^b^	0.129 ± 0.02 ^b^	0.186 ± 0.10 ^b^	0.161 ± 0.09 ^a^	0.016 ± 0.01 ^b^
*p*		<0.001	<0.001	<0.001	<0.001	<0.001	<0.001	<0.001
Wilting								
Fresh forage	24	0.928 ± 1.48	1.033 ± 1.59	0.056 ± 0.07	0.369 ± 0.36	0.165 ± 0.13	0.108 ± 0.07	0.046 ± 0.07
Wilted forage	24	1.166 ± 1.90	1.349 ± 2.32	0.061 ± 0.08	0.363 ± 0.41	0.223 ± 0.25	0.142 ± 0.13	0.058 ± 0.10
*p*		0.220	0.140	0.647	0.907	0.076	0.116	0.220
Sampling								
1.	16	1.278 ± 2.06	1.413 ± 2.34	0.069 ± 0.09	0.430 ± 0.48	0.212 ± 0.26	0.136 ± 0.13	0.064 ± 0.10
2.	16	1.153 ± 1.82	1.200 ± 1.98	0.061 ± 0.08	0.360 ± 0.37	0.200 ± 0.18	0.128 ± 0.09	0.058 ± 0.09
3.	16	0.710 ± 1.10	0.960 ± 1.64	0.045 ± 0.05	0.308 ± 0.28	0.170 ± 0.15	0.110 ± 0.08	0.036 ± 0.06
*p*		0.050	0.226	0.149	0.168	0.556	0.596	0.050
Study plot								
Vatín	24	1.180 ± 1.82	1.421 ± 2.30 ^a^	0.060 ± 0.08	0.339 ± 0.32	0.202 ± 0.17	0.128 ± 0.09	0.059 ± 0.09
Troubsko	24	0.914 ± 1.59	0.960 ± 1.60 ^b^	0.057 ± 0.08	0.393 ± 0.44	0.186 ± 0.22	0.122 ± 0.12	0.046 ± 0.08
*p*		0.172	0.034	0.701	0.300	0.628	0.755	0.184

Statistically significant differences (*p* < 0.05) between means are indicated by different superscripts (^a,b^) within columns.

**Table 3:** Total isoflavone content in forage of *Trifolium pratense*, *Medicago sativa*, *Trifolium resupinatum*, and *Trifolium alexandrinum* (% of dry matter).

Species	Overall Representation of Isoflavones
*Trifolium pratense* L.	1.001
*Medicago sativa* L.	0.056
*Trifolium resupinatum* L.	0.061
*Trifolium alexandrinum* L.	0.094
